# Enhancing image based classification for crop disease detection using a multiclass SVM approach with kernel comparison

**DOI:** 10.1038/s41598-025-23568-w

**Published:** 2025-11-17

**Authors:** Parkavi Sridhar, Parthiban Angamuthu

**Affiliations:** Department of Mathematics, School of Advanced Sciences, Vellore Institute of Technology, Vellore, Tamil Nadu 632 014 India

**Keywords:** Machine learning (ML), Image preprocessing, Bilateral filter (BF), Augmentation, Image segmentation, Feature extraction, Multiclass SVM kernels, Plant disease classification, Mathematics and computing, Imaging

## Abstract

Agricultural production is still quite susceptible to plant diseases, despite the fact that it is essential to both economic growth and food security. Yellow rust can lower wheat yields by 20–30%, red rust by 5–10%, and anthracnose by up to 60% in crops including cotton and mango. For losses to be minimized, early and precise detection is therefore crucial. Preprocessing, segmentation, feature extraction, and classification are all included in this study’s machine learning-based framework for detecting various crop leaf diseases. To test the model, 9,111 carefully chosen images that were balanced through augmentation were employed. The novelty of this work lies in combining bilateral filtering and GraphCut segmentation with texture-based feature extraction and a systematic comparison of multiclass SVM kernels across a multi-crop dataset. Experimental results using stratified 5-fold cross-validation show that the linear kernel SVM achieved the best performance, with 99.0% accuracy, 98.6% precision, 98.7% recall, and 98.6% F1-score–outperforming earlier SVM-based approaches. These findings demonstrate the effectiveness of kernel selection and preprocessing in enhancing disease classification and provide a strong basis for future comparisons with deep learning methods to build scalable and reliable plant disease detection systems.

## Introduction

Agriculture is vital for economic growth and living standards, with the food processing sector playing a key role in enhancing product exports. In developing nations, food processing grows due to export income and domestic demand. Pest infestations are a major concern, damaging crops and lowering market value. Addressing pests, pathogens, and weeds is crucial for maintaining productivity and quality, as insects significantly impact crop yields. Effective monitoring of insect-related losses is essential for ensuring agricultural safety and profitability.

In India, plant diseases such as Bacterial Leaf Blight, Rice Blast, Rusts, and Powdery Mildew heavily impact crops, cutting yields by up to 50% for rice, potatoes, sugarcane, tomatoes, bananas, and citrus. These diseases pose a threat to food security, increase management expenses, and lead to greater environmental damage from chemical use. In 2024, addressing these challenges requires implementing effective disease control, developing resistant crop varieties, and adopting better farming practices to safeguard agricultural productivity and economic stability^1^. India’s major crops, such as cotton, cashew, sugarcane, and wheat, are enhanced by cutting-edge technologies to improve both productivity and sustainability. Deep learning aids in disease management for cotton, while precision agriculture supports cashew farming. Integrated pest control and drone technology optimize sugarcane production, and wheat cultivation benefits from disease-resistant varieties and precision farming methods to secure food production. Recent developments in deep learning (DL) and machine learning (ML) have made it possible to automatically identify diseases from leaf images. In the field of illness recognition, convolutional neural networks (CNNs), support vector machines (SVMs), and hybrid models have demonstrated encouraging outcomes. Three factors, however, limit the majority of current research: (i) they frequently concentrate on single-crop datasets; (ii) they do not systematically assess kernel functions in SVM-based classification; and (iii) preprocessing and segmentation techniques are either ignored or just briefly discussed. Existing models’ robustness and generalizability are diminished by these constraints.

Akinosun et al.^2^ investigated the use of AI techniques, including DL and ML, to identify wheat rust diseases. They tested SVM, CNN, and a combined CNN&ViT model on the wheat rust classification dataset. The CNN&ViT model achieved the highest accuracy at 98.3%, outperforming CNN, which had an accuracy of 95.97%. SVM achieved a precision of 91.7%. All models performed well, with precision, recall, and F1-scores exceeding 90%, proving effective for classifying small to medium-sized image datasets.

Ahmed et al.^3^ investigated the use of GLCM-based textural features for classifying crops with greyscale images. They demonstrated that features such as energy, dissimilarity, and angular second moment could achieve up to 97.5% accuracy using machine learning techniques like Naive Bayes, random forests, SVMs, and neural networks. However, DL models like CNNs performed less effectively, with accuracies of 50.82% for greyscale and 25% for textural images. Data collection faced challenges due to drone flight height restrictions. Future improvements are anticipated through higher-altitude data collection and more advanced surface feature analysis. Data preprocessing, exploratory data analysis, and detection are the three steps of the plant disease diagnosis approach developed by Kumar et al.^4^, Sardarkrushinagar Dantiwada Agricultural University in Gujarat, India provided them with real-time data from satellite sources and soil sensors. They created a machine learning model by evaluating the data to derive insights, and this model outperformed previous methods with an accuracy of over 98%. For quickly identifying plant diseases, this approach provides an economical and successful approach.

Plants, which supply over 80% of human nutrition, are crucial for global food security. Neelakantan et al.^5^ study addressed the threat posed by plant diseases and evaluated machine learning algorithms for detecting them. Techniques such as RF, SVM, DT, KNN, and NB were tested alongside image processing methods. RF demonstrated the best performance with an accuracy of 89%, highlighting its potential over traditional, error-prone methods.

A machine learning framework for crop leaf disease detection is presented in this work, addressing existing constraints in the field by integrating crucial steps such as preprocessing, segmentation, feature extraction, and classification. 9,111 leaf images of different crops made up the heterogeneous collection, which was balanced using data augmentation techniques and gathered from publicly accessible sources. GraphCut and a Y*Cb*Cr color space were employed to segregate sick areas, and bilateral filtering was applied to improve image quality during preprocessing. Gray-Level Co-occurrence Matrix (GLCM) and Local Binary Pattern (LBP) were then used to extract texture features. A multiclass SVM with four distinct kernels–Linear, RBF, Quadratic, and Cubic–was then used to classify the disease.

This work’s main contributions are as follows:

- Dataset construction and augmentation: 9,111 images from open-access repositories were compiled into a balanced, multi-crop dataset.

- Implementing a single workflow that combines texture-based feature extraction, segmentation, and image enhancement is known as integrated pipeline design.

- Evaluation of SVM kernels: Using stratified 5-fold cross-validation, four SVM kernels are compared and evaluated based on a number of performance metrics, such as accuracy, precision, recall, and F1-score.

This paper focuses on the systematic assessment of SVM kernel performance inside a comprehensive pipeline applied to a newly curated dataset, rather than presenting SVM as a novel approach. The findings are intended to promote repeatability and provide a strong foundation for the subsequent deep learning model development.

Despite these advancements, several challenges still persist. Many existing studies focus on single-crop or single-disease datasets, which limits the ability to generalize the models to broader agricultural contexts. In SVM-based approaches, a thorough evaluation of different kernel functions is often neglected, with most studies relying on just one kernel. Additionally, preprocessing and segmentation–key steps for improving classification accuracy–are often oversimplified or not fully integrated into the overall pipeline.

This study is innovative because it combines hybrid texture-based feature extraction techniques (GLCM and LBP) with bilateral filtering and GraphCut segmentation. All of these techniques are tested on a meticulously selected and enhanced multi-crop dataset. Our study adopts a more thorough strategy by methodically comparing different kernels (linear, RBF, quadratic, and cubic) within a single pipeline, in contrast to earlier SVM-based methods that concentrated on a small number of datasets. This combination makes the model for agricultural disease identification more robust, generalizable, and applicable in the real world.

Existing algorithms continue to have several drawbacks despite their encouraging outcomes. The majority of earlier studies only used records from a particular crop or illness, which restricts their applicability in different agricultural environments. Usually, SVM-based research simply assesses one kernel without doing a thorough comparison. Preprocessing and segmentation are two crucial processes that are frequently neglected or simplified, which lowers accuracy in practical settings. To overcome these limitations, our method employs a hybrid GLCM and LBP feature extraction approach on a curated and enhanced multi-crop dataset, utilizing bilateral filtering and GraphCut segmentation. Additionally, we compare many SVM kernels (linear, RBF, quadratic, and cubic) in a methodical manner using the same pipeline, showing enhanced robustness, reproducibility, and usefulness.

## Related works

Hou et al.^6^ proposed an automatic graph-cut algorithm to segment potato leaf images, extracting texture features using Local Binary Patterns (LBP) and color features from the Lab* color space. They evaluated four classifiers kNN, SVM, ANN, and RF for identifying potato diseases. The segmentation achieved a mean Intersection over Union (IoU) of 93.70%. Among the classifiers, the SVM performed best, with a precision of 92.1%, using a combination of LBP and color histograms from the Lab* color channels. Pantazi et al.^7^ developed a computerized system for agricultural disease detection using LBP for feature extraction and binary classification. Applied to various crop leaf images, the system achieved a 95% success rate across 46 plant-condition pairings, demonstrating strong adaptability, particularly with vine leaves.

Kasinathan et al.^8^ developed a pesticide detection algorithm utilizing foreground extraction and contour identification to classify insects in complex backgrounds from datasets such as Wang, Xie, Deng, and IP102. They employed classifiers like CNN, ANN, SVM, KNN, and NB with 9-fold cross-validation, focusing on shape features. The CNN model performed best, achieving classification rates of 91.5% for nine insect classes and 90% for twenty-four classes. Pallathadka et al.^9^ introduced a machine learning approach for classifying and detecting leaf diseases using SVM, NB, and CNN models. They enhanced accuracy through image processing techniques such as histogram equalization for noise reduction, K-means clustering for image segmentation, and PCA for feature extraction.

Nyasulu et al.^10^ explored the classification of tomato fungal leaf diseases using ANNs, KNN, RF, and SVM, leveraging GLCM texture features. The ANN model achieved the highest performance, with 94% accuracy and Precision, Recall, and F1-scores near 93.8%. The study underscores the value of GLCM features in disease detection and recommends further enhancement through CNN technologies. Nalini et al.^11^ explored the use of ML to predict crop diseases based on temperature data, comparing the performance of the KNN and Max Voting techniques on a dataset of 500 samples. The Max Voting method outperformed KNN, achieving 91% accuracy compared to KNN’s 88%, and demonstrated a lower RMSE (3.97 versus KNN’s 4.34), making it the more effective method for temperature-based crop disease prediction.

Using data from 12 crop species and 17 diseases included in the “Plant Village” dataset, Ahmed et al.^12^ investigated a range of deep learning and machine learning techniques for plant disease identification. A 99% accuracy rate was achieved by using methods like CNNs, GLCMs, and SVMs. A variety of criteria were used to evaluate the models’ performance, including accuracy, precision, recall, and F1-score. CNNs outperformed KNNs in terms of execution. Sobiyaa et al.^13^ used a dataset of 3,671 annotated photos and a smartphone camera to construct a ML system for paddy disease classification. The system used Deep-CNN to extract important illness features while classifying images using the Inception v3 model using CNN and TensorFlow. With scores of 91.9% for blast, 93.2% for bacterial blight, 89% for sheath rot, and 90% for brown spot, the DNN approach showed excellent accuracy. The system’s performance was assessed using critical performance measures as TPR, F1-score, precision, and accuracy.

In their ML models for grape leaf disease detection, Shantkumari et al.^14^ focused on the Convolutional Neural Network-based Classification (CNNC) model and the Improvised K-Nearest Neighbour (IKNN) model. They extracted pertinent features from the Plant Village Dataset using pixel encoding approaches, using GPOR to record interactions between distant pixels and CIDOR for effective histogram representation. Compared to the CNNC model, the IKNN model had better classification accuracy. Hybrid ML techniques were created by Suresh et al.^15^ to identify and classify groundnut leaf diseases in real time (GLD-HML). They applied the multi-objective sunflower optimization (MSO) algorithm for the best feature selection and the Improved Crow Search (ICS) technique to segment the leaf disease areas. Multiple disease types were classified using a deep neural network based on moth optimization (MO-DNN), and the IoT idea was combined to promptly send results to farmers via mobile devices. GLD-HML significantly outperformed previous techniques in terms of accuracy, precision, F-measure, and recall when evaluated against standard datasets.

In agricultural AI, recent studies have looked at both wide-ranging applications and segmentation-focused approaches. A thorough analysis of AI methods in pre-harvesting and post-harvesting phases revealed both potential and persistent problems, including deployment problems and dataset constraints^16^. The importance of precise segmentation in enhancing disease analysis was also shown by a segmentation-based framework for estimating the severity of disease in infected leaves^17^. Although this research offers important insights, they are either focused mainly on deep learning or have a broad perspective. By comparison, our study presents a unified pipeline that systematically assesses SVM kernels on a curated multi-crop dataset, integrating preprocessing, segmentation, hybrid feature extraction, and kernel-based classification.

**Table:** 

S.No	Reference	Methodology	Dataset	Data samples	Subjects	Feature Extract	Contribution	Limitations
1	Kasinathan et al.^8^	ANN, SVM, KNN, NB & CNN	Wang, Xie, Deng, and IP102	2,706	24	Shape	To boost the effectiveness of the classification models, 9-fold cross-validation was used	Assessed on a specific set illnesses and put into practice with custom features
2	Nyasulu et al.^10^	ANNs, KNN, RF & SVM	Custom built	11250	4	GLCM	Detection and distinction of these illness utilizing GLCM-based features to get the maximize accuracy via ANN	Assessed a small group of individuals and require to investigate CNN grouping
3	Nalini et al.^11^	KNN and Max Voting methods	Kaggle crop	500	2	Demands are used as traits	Enhance the precision and lower the RMSE, KNN, and Max Vote approaches used to forecast illnesses depending on temperature	In a controlled or uncontrolled setting, created weights can be utilized again to identify crop illness
4	Ahmed et al.^12^	SVM, CNN	Plant Village	8350	9	GLCM	Robotic gadgets can be used to diagnose diseases and save time tracking on huge crop fields	Merging CNN with drone-captured aerial photos to find objects
5	Sobiya et al.^13^	Tensor flow Initialization v3 model, including CNN	uniquely constructed	3,671	5	Deep-CNN	Quick disease categorization and detection, CNN is used in the TensorFlow Inception v3 model	specific subjects and activities were used in the test
6	Shantkumar et al.^14^	Accurately recognize grape leaf diseases, use the CNNC & IKNN	Plant Village	4,062	4	pixel-by-pixel histogram	increase the classification accuracy of leaf diseases for effective detection	precise techniques applied to the widely used designs
7	Suresh et al.^15^	GLD-HML, ICS, MSO, MO-DNN	SMFD	129	6	MSO	automated identify and categorize method utilizing GLD-HML that is IoT-based	tested with a limited group of participants and put into practice with custom features
8	Akinosun et al.^2^	SVM, CNN, CNN&ViT	WRCD	3,679	3	ViT’s and CNN	CNN converges quickly, but combining CNN with ViT continues to show improvements with more training epochs	ViT on smaller datasets is that it needs a lot of data to perform well
9	Kiran et al.^18^	SVM, Deep CNN	Plant Village	54,000	5	SVM	To increase training efficacy and reliability, SVM and deep CNN were employed	When examining the particular dataset used, greater dataset values will yield more accurate results
10	Hou et al.^19^	Otsu thresholding, color statistical thresholding, SVM, KNN, ANN, RF	AI Challenger Global AI Contest	2840	5	LBP	approach is made to precisely determine the kind and severity of infection	Intend to use DL in the future to attain faster training times and higher accuracy

## Dataset description

Images from the “Crop Pest & Disease Detection dataset” and the “20k+ Multi-Class Crop Disease Images dataset” Kaggle databases were used in the proposed study^20,21^. Anthracnose, red rust, yellow rust, and healthy were the four classes that were the focus of a carefully selected subset that was produced from these sources. 9,111 images were used in all, including 2,474 healthy samples, 3,054 red rust, 1,006 yellow rust, and 2,577 anthracnose images. The images, taken from different angles, distances, and lighting conditions, offer high-resolution information and present realistic recognition job problems. Additionally, pictures of healthy plants were added as a reference class. An unprocessed sample image from the dataset is shown in Fig. 2. Table 1 lists the class distribution used in this study. The dataset exhibits class imbalance, with red rust being over represented compared to yellow rust and anthracnose. The crops that were chosen are cashew, cotton, sugarcane, and wheat, all of which are extensively grown in India and a number of other nations.

**Table 1:** Data description of various crops.

S.No	Disease	Dataset	Types of crops	Pixel size	No of images before augmentation	No of images after augmentation
1	Antracnose	^21^	Cashew, Cotton	224$\times$224	2,577	2500
2	Red Rust	^21^	Cashew, Sugarcane	224$\times$224	3,054	2500
3	Yellow Rust	^20^	Sugarcane, Wheat	224$\times$224	1,006	2500
4	Healthy	^20^	Cashew, Sugarcane, Wheat	224$\times$224	2,474	2500

## Proposed method

**Fig. 1:**
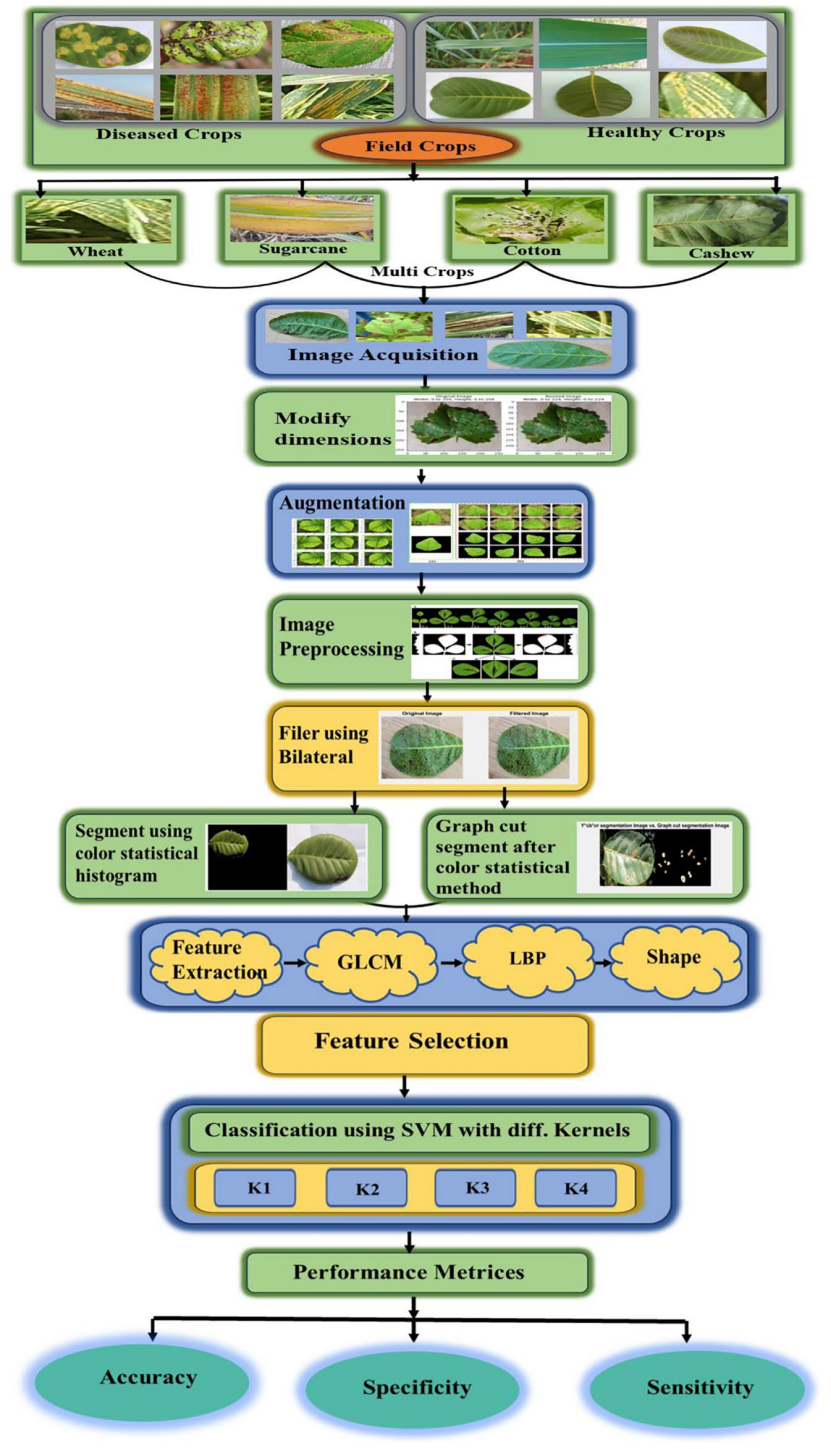
Proposed methodology.

The five primary steps of the suggested framework (Fig. 1) are (i) image acquisition, (ii) preprocessing, (iii) segmentation, (iv) feature extraction, and (v) classification. 9,111 carefully selected multi-crop leaf images were scaled to 224$\times$224 pixels and then balanced using augmentation. Bilateral filtering and background removal were used throughout the preprocessing phase to enhance the quality of the images. The GraphCut method was used to segment the Y*Cb*Cr color space in order to identify unhealthy areas. Local Binary Pattern (LBP) and Gray-Level Co-occurrence Matrix (GLCM) descriptors were then used to extract texture information. Finally, disease classification was carried out with a multiclass SVM using four kernel functions - linear, RBF, quadratic, and cubic. Performance was evaluated using accuracy, precision, recall, specificity, and F1-score, with stratified 5-fold cross-validation ensuring reproducibility. The following subsections describe each stage of the pipeline in detail.

### Image acquisition

**Fig. 2:**
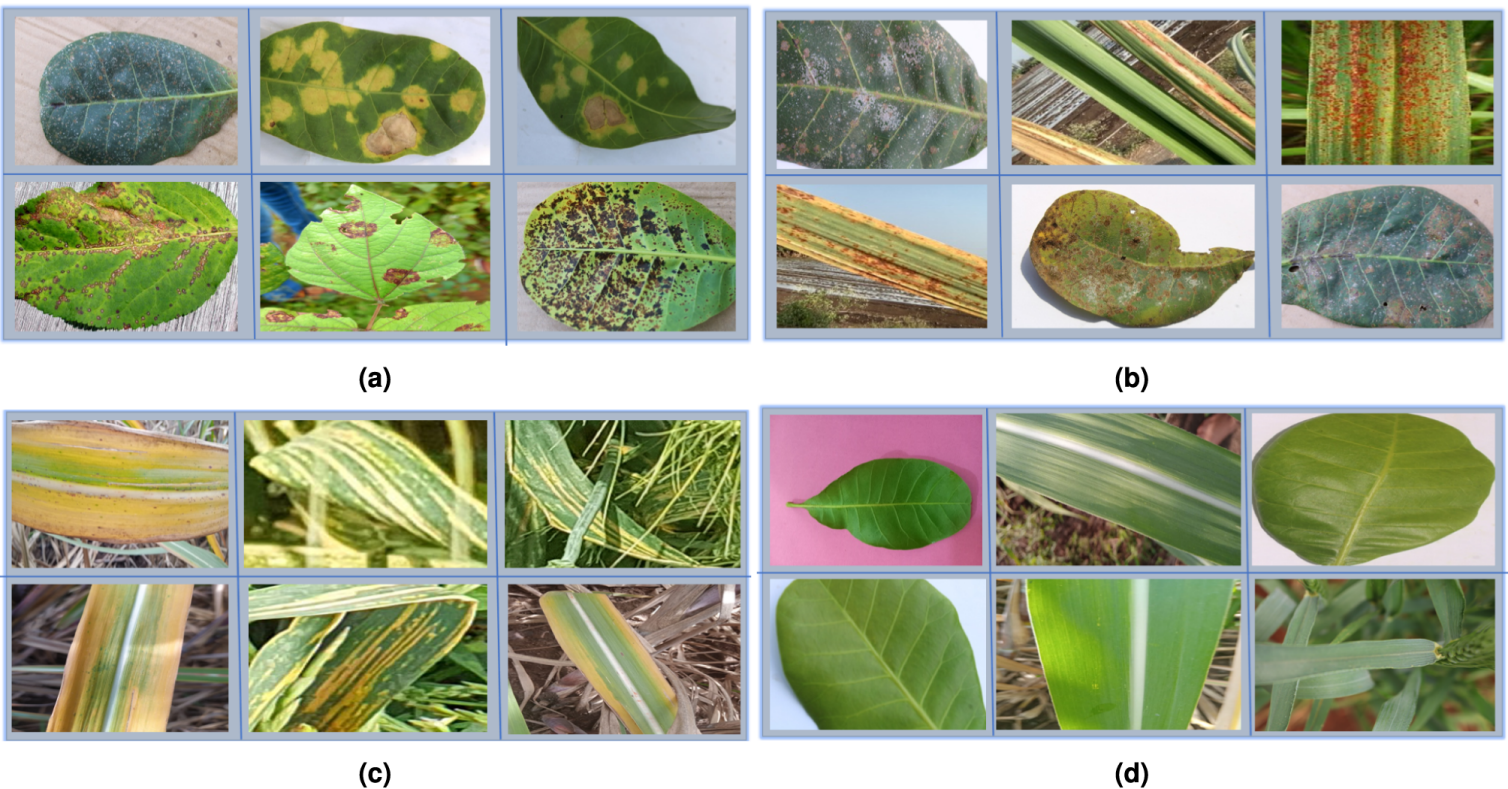
Input crop images (**a**) Antracnose (**b**) Red rust (**c**) Yellow rust (**d**) Healthy.

The proposed method begins with the acquisition of images. These images are obtained from the internet or captured using a digital camera. A total of 9,111 picture samples are available for the three groups of rust diseases such as yellow rust, red rust, and anthracnose that are primarily found on crops such as cotton, wheat, cashew, sugarcane, barley, and others. Anthracnose, a fungal disease affecting a variety of plants, is influenced by plant species, fungus type, and weather conditions. It is more prevalent in warm, humid climates. Red rust, another fungal threat, also thrives in warm, humid conditions, but its impact varies depending on the specific plant, the type of fungus, and the overall weather. Yellow rust affects cereal crops like wheat, barley, and triticale, particularly under damp, cool conditions with high humidity and rainfall. The images have been resized to dimensions of 224$\times$ 224 pixels. In Fig. 2, depicts the sample images of both diseased and healthy leaves.

### Image preprocessing

**Fig. 3:**
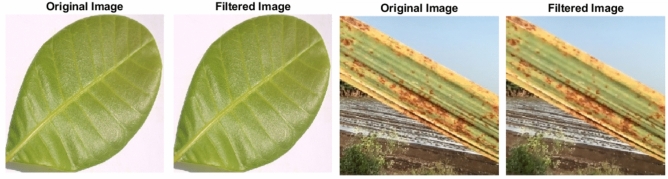
Image preprocessing using bilateral filter.

Preprocessing was applied to enhance image quality by removing background noise and irrelevant details. A bilateral filter was used to reduce noise while preserving edges, ensuring disease boundaries remained clear for accurate segmentation^22^. Morphological operations were employed to remove small artifacts and shadows. The original and noise-reduced images are shown in Fig. 3.

### Bilateral filter

The bilateral filter is a non-linear, edge-preserving smoother that averages neighboring pixels using both spatial proximity and intensity similarity, thereby reducing noise while retaining sharp disease boundaries important for segmentation^23^. We apply it prior to segmentation (see Fig. 3). The filtered intensity at pixel *u* is

$$I_f(u)=\frac{1}{W_u}\sum _{v\in S} I(v)\, \exp \!\left( -\frac{\Vert u-v\Vert ^2}{2\sigma _s^2}-\frac{\Vert I(u)-I(v)\Vert ^2}{2\sigma _r^2}\right) ,$$

where $W_u$ normalizes the weights, and $\sigma _s,\sigma _r$ control spatial and range smoothing, respectively.

### Augmentation

To address class imbalance and reduce overfitting, additional images were generated using Roboflow. Augmentation techniques included rotations (clockwise and counterclockwise) and horizontal/vertical flips, expanding the dataset from 9,111 to 10,000 images (Table 1).

## Segmentation

Segmentation was performed in two stages: first separating the leaf from the background, and then distinguishing healthy from diseased tissue. The Y*Cb*Cr color space, which separates luminance (Y) from chrominance components (Cb, Cr), was employed as it provides better discrimination than the RGB model^24^. The GraphCut method was then applied in this color space to accurately localize diseased regions by formulating segmentation as an energy minimization problem^25^.

### GraphCut method

GraphCut is a widely used segmentation technique that models an image as a graph, where each pixel is a node and edges represent similarity or connectivity^26^. The method formulates segmentation as an energy minimization problem combining a data term, which measures how well a pixel fits the foreground or background, and a smoothness term, which enforces spatial coherence between neighboring pixels. The steps for implementing this method are outlined in Algorithm 1. The general form of the energy function is:

$$\begin{aligned} E(\kappa ) = \sum _i D(\kappa _i) + \sum _{(i,j)\in N} V(\kappa _i, \kappa _j) \end{aligned}$$

where $D(\kappa _i)$ is the data term for pixel *i*, $V(\kappa _i, \kappa _j)$ is the smoothness penalty between neighboring pixels, and *N* is the set of adjacent pixel pairs.

**Fig. 4:**
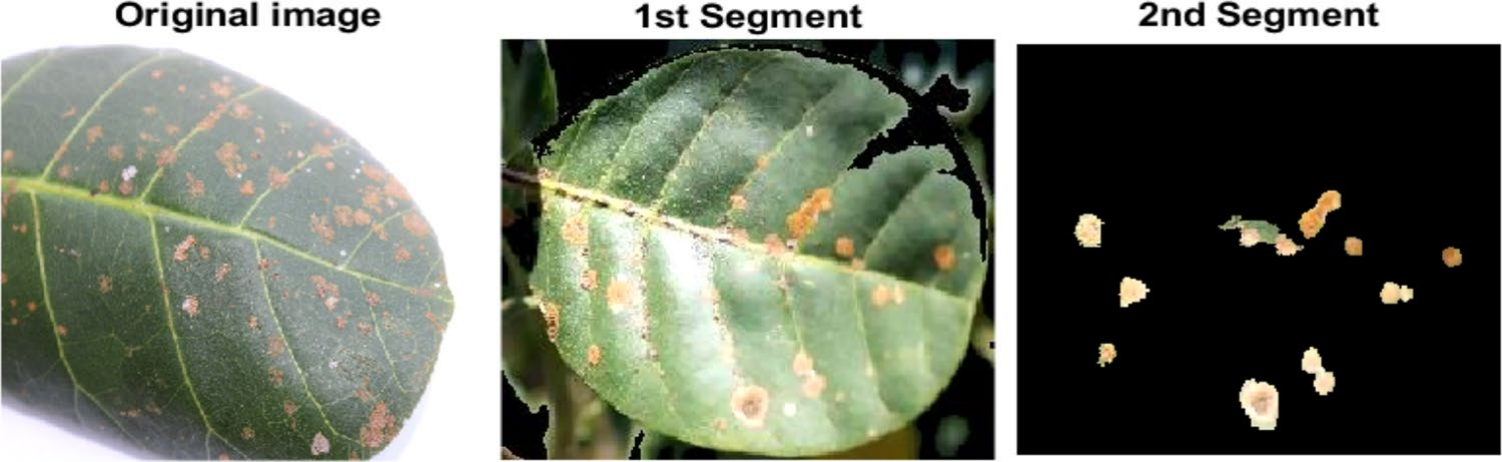
Two-step segmentation to identify the location of disease.

In practice, GraphCut leverages Gaussian Mixture Models (GMMs) to model foreground and background distributions, iteratively refining the segmentation until convergence^6^. By combining color statistics with spatial relationships, the method enables accurate localization of diseased leaf regions. Its effectiveness has been demonstrated in plant disease studies, including leaf segmentation tasks^27^. A sample segmentation output is shown in Fig. 4.

The segmentation process using GraphCut can be summarized as:

1. Initialize foreground and background regions in the Y*Cb*Cr color space using color statistics.
2. Construct a graph $G=(V,E)$ with pixels as nodes and edge weights based on color similarity and spatial proximity.
3. Model foreground and background using Gaussian Mixture Models (GMMs).
4. Iteratively update GMMs and pixel labels through GraphCut optimization until convergence.

**Algorithm 1:**
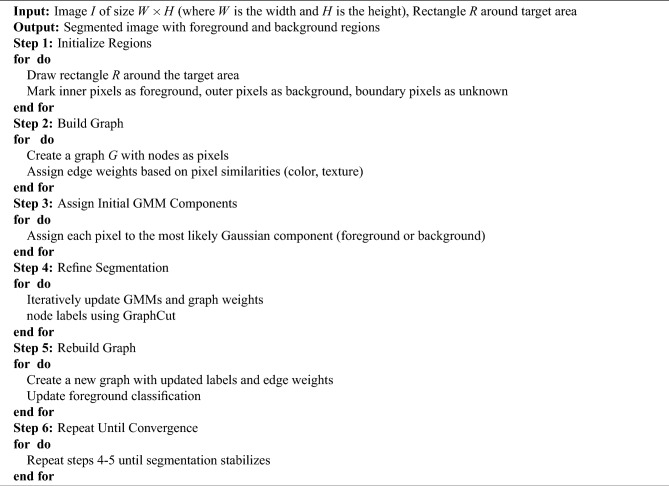
Pseudo code: Image Segmentation Using GraphCut

### Feature extraction

Feature extraction was performed using a hybrid approach combining shape and texture descriptors. Discrete Wavelet Transform (DWT) was first applied to reduce image size and capture frequency-domain information. To enhance robustness, texture features were extracted using the Gray-Level Co-occurrence Matrix (GLCM) and Local Binary Patterns (LBP), which are widely used in plant disease recognition tasks^28,29^. GLCM captures spatial intensity relationships through measures such as contrast and homogeneity, while LBP encodes local texture patterns that are resistant to illumination changes. The computed features from DWT sub-bands were further evaluated using Euclidean and Canberra distances to provide similarity measures^30^.

#### Gray-Level Co-Occurrence Matrix (GLCM)

The Gray-Level Co-Occurrence Matrix (GLCM) is a statistical method widely used to describe the spatial relationship between pixels in an image^31^. Each entry $\rho (i,j)$ of the GLCM represents the probability of two pixels with gray levels *i* and *j* occurring together in a specified spatial relationship (e.g., horizontal, vertical, or diagonal). By analyzing these co-occurrence probabilities, texture features can be derived that are highly informative for disease classification.

From the GLCM, several first- and second-order statistical features are computed:

- **Skewness:** Measures asymmetry in the intensity distribution:
  $$Skewness = \dfrac{n}{(n-1)(n-2)} \sum \dfrac{(x_i-\overline{x})^3}{s}$$
  where *n* is the number of observations, $x_i$ is the pixel intensity, $\overline{x}$ is the mean, and *s* is the standard deviation.
- **Kurtosis:** Quantifies “tailedness” of the distribution:
  $$\begin{aligned} Kurtosis = \dfrac{n(n+1)}{(n-1)(n-2)(n-3)} \sum \dfrac{(x_i-\overline{x})^4}{s} - \dfrac{3(n-1)^2}{(n-2)(n-3)} \end{aligned}$$
- **Contrast:** Captures local variations in gray levels:
  $$\begin{aligned} S_c = \sum _{i}\sum _{j}(i-j)^2 \rho (i,j) \end{aligned}$$
- **Correlation:** Measures the linear dependency of gray levels:
  $$\begin{aligned} S_o = \dfrac{\sum _{i}\sum _{j} (ij)\rho (i,j) - \psi _x \psi _y}{\tau _x \tau _y} \end{aligned}$$
  where $\psi _x, \psi _y$ are mean gray levels and $\tau _x, \tau _y$ their standard deviations.
- **Variance:** Quantifies dispersion of intensity values:
  $$\begin{aligned} S_V = \sum _{i,j=1}^N (i-j)^2 \rho (i,j) \end{aligned}$$
- **Cluster Shade:** Measures asymmetry in the co-occurrence matrix:
  $$\begin{aligned} S_{cs} = \sum _{i,j=1}^N (i-M_x+j-M_y)^2 \rho (i,j) \end{aligned}$$
  where $M_x, M_y$ are the means of gray levels.
- **Homogeneity:** Assesses texture uniformity:
  $$\begin{aligned} Homogeneity = \sum _{i=0}^{N-1} \sum _{j=0}^{N-1} \frac{\rho (i,j)}{1+|i-j|} \end{aligned}$$
- **Energy:** Reflects textural uniformity:
  $$\begin{aligned} S_N = \sum _{b=0}^{L-1} [\rho (b)]^2 \end{aligned}$$
- **Entropy:** Measures randomness in texture:
  $$\begin{aligned} Entropy = -\sum _{i=1}^{\rho }\sum _{j=1}^{\rho } P(i,j)\log P(i,j) \end{aligned}$$
  where *P*(*i*, *j*) is the normalized co-occurrence matrix entry.

These features collectively describe intensity distribution (first-order statistics) and spatial relationships (second-order statistics), providing robust texture descriptors for plant disease classification.

#### Local Binary Pattern (LBP)

Local Binary Pattern (LBP) is a widely used texture descriptor that assigns binary codes to pixels by thresholding each pixel against the values of its neighborhood. Typically applied on a $3 \times 3$ grid, this process generates up to $2^8 = 256$ unique labels per patch^32,33^. The main advantages of LBP are its robustness to illumination changes and computational efficiency, making it effective for real-time applications. Variants such as rotation-invariant and multiscale LBP further enhance its ability to capture diverse texture characteristics. In plant disease recognition, LBP has been particularly effective in characterizing fine-grained leaf texture patterns.

## Multiclass SVM-based classification techniques

Support Vector Machine (SVM), introduced by Boser, Guyon, and Vapnik in 1992, is a supervised learning method widely used for binary classification and regression^34^. The main strength of SVM lies in its ability to handle both linear and nonlinear classification problems. Kernel methods allow nonlinear data to be mapped into higher-dimensional space, where SVM finds the optimal separating hyperplane by maximizing the margin between classes.

In simple terms, SVM learns the best hyperplane that separates classes with the maximum margin. By using kernels for nonlinear data, it balances accuracy and complexity through regularization. SVM is effective in high-dimensional spaces, although it can be computationally demanding. For multiclass problems, this study applies the “one-vs-all” strategy, where multiple binary classifiers are trained and their outputs combined. When applied to four-group classification tasks, SVM, a popular machine learning technique, delivers significant results. In multiclass tasks, the problem is divided into multiple binary classification tasks, and the results from these SVM classifiers are subsequently combined^35^.

The final stage of this work is classification. A multiclass SVM is used to evaluate the proposed method by categorizing different leaf diseases. This phase uses feature vectors and their corresponding classes from the training dataset as inputs, while the outputs are the classifications of the disease type impacting the leaf. Since kernel choice strongly affects classification, this study evaluates and compares four kernels–linear, RBF, quadratic, and cubic.

### Experimental setup

Experiments were performed using stratified 5-fold cross-validation, ensuring balanced class distributions in each fold. A fixed random seed was applied for reproducibility. Performance was evaluated using accuracy, precision, recall, and F1-score, reported as mean ± standard deviation across the folds.

### Linear kernel

A specific type of SVM designed for linearly separable data is the Linear Kernel SVM. It identifies the best hyperplane to partition data points into separate groups. The Linear SVM is an effective and intuitive kernel method that creates a linear decision boundary for linearly separable data and works well with large datasets. However, linear SVMs face challenges with non-linearly separable datasets, as outliers can significantly impact the location of the optimal hyperplane^36^. The mathematical description of the linear kernel function is:

$$\begin{aligned} \mathscr {K}(q, s) = q^T * s \end{aligned}$$

where, $\mathscr {K}(q, s)$ is the kernel function, *q* and *s* are input data points, *T* denotes transpose.

### Radial Basis Function (RBF) kernel

In SVMs, the RBF kernel manages non-linear interactions by implicitly transforming input data into a higher-dimensional space, allowing for linear separation^37^. The RBF kernel function is mathematically described as:

$$\begin{aligned} \mathscr {K}(\Upsilon ,\Upsilon ')=exp(-\gamma ||\Upsilon _{i}-\Upsilon _{j}||^{2}) \end{aligned}$$

where, $\Upsilon _{i}$ and $\Upsilon _{j}$ are data points, $||\Upsilon _{i}-\Upsilon _{j}||^{2}$ squared euclidean distance, and $\gamma$ is Kernel parameter controlling the spread.

### Cubic kernel

The cubic kernel is a type of polynomial kernel function frequently utilized in machine learning models, including SVMs. It transforms the input data into a higher-dimensional space, enabling the detection of non-linear decision boundaries^38^. The cubic kernel is defined by the following mathematical formula:

$$\begin{aligned} \mathscr {K}(\Upsilon _{i},\Upsilon _{j})=(\Upsilon _{i}.\Upsilon _{j}+c)^{3} \end{aligned}$$

Here, $\Upsilon _{i}$ and $\Upsilon _{j}$ are feature vectors, and $\Upsilon _{i} \cdot \Upsilon _{j}$ indicates their dot product. The constant $c$ is added to the dot product before it is cubed.

### Quadratic kernel

A quadratic kernel SVM uses a degree-2 polynomial kernel to map input features into a higher-dimensional space, enabling the creation of quadratic decision boundaries^39^. It captures more complex patterns than a linear kernel while maintaining computational efficiency. Key hyperparameters include the regularization parameter $C$ and the kernel constant $c$. The polynomial kernel of degree 2 is mathematically expressed as:

$$\begin{aligned} \mathscr {K} (\Upsilon _{i},\Upsilon _{j})=(\langle \Upsilon _{i},\Upsilon _{j} \rangle )+ c^{2} \end{aligned}$$

### Two category classification (healthy and defective)

**Fig. 5:**
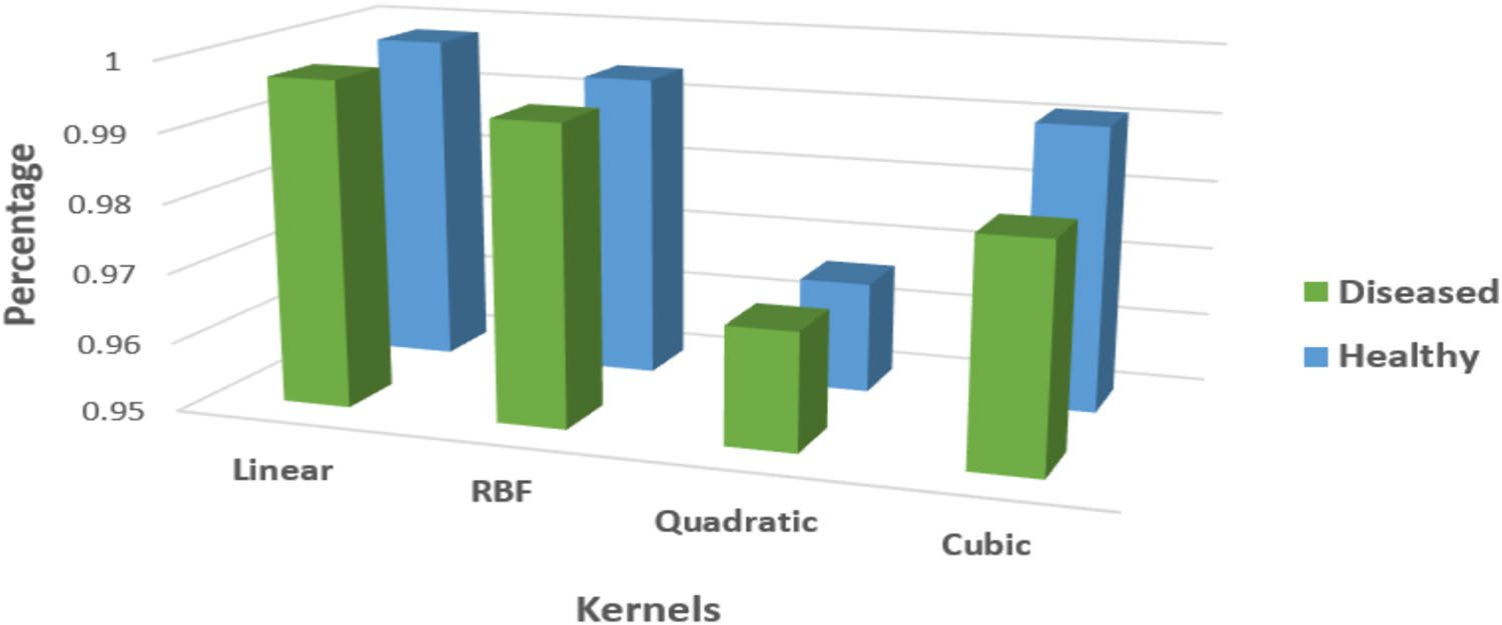
Classification of diseases into two groups.

For classifying different diseases, a two-category classification approach creates one group for healthy samples and another for diseased ones. This approach addresses challenges related to classification procedures and data collection. Consequently, two-category classification is introduced initially. We conduct various tests for two-category classification, using each classifier to categorize the diseases. Disease grading is accomplished using tailored classification models. The experiment’s greatest precision rates are shown in Fig. 5. Table 2 displays the maximum accuracy rate of 99.0, which is produced by the SVM employing a linear kernel, and the precision on a healthy leaf is 99.0%; however, on a faulty leaf it is (98.2%. Table 3 presents the accuracy results for the binary classification performance, which differentiates between healthy and diseased cases across the four tested kernels.

### Multi-category classification

A comprehensive disease classification system should offer more detailed categorization beyond simply identifying whether leaves are healthy or diseased. Implementing a multicategory classification involves manually sorting leaves into four categories (healthy, red rust, yellow rust, and anthracnose) and several crops (cotton, wheat, sugarcane, and cashew).

Antracnose

Dark stains on plants, along with damage and decreased yield, result from the fungal disease anthracnose. Control measures include the use of fungicides, promoting air movement, maintaining sanitation, and employing resistant varieties.

Red rust

Wheat is affected by reddish-brown pustules known as red rust, caused by Puccinia fungi. The disease spreads via the wind and thrives in mild, wet environments. Control measures include the use of fungicides, crop rotation, resistant cultivars, and early identification.

Yellow rust

Yellow rust, caused by Puccinia striiformis, infects wheat with yellow pustules. It thrives in cool, moist conditions and spreads through the wind. Control measures include the use of resistant varieties, fungicides, and early detection.

A distinct classifier is used for multi-category (4-class) grade. We look through every classifier’s output. Similar to the four-category findings, SVM with a linear kernel has the maximum accuracy rate of 99.0%, revealing that this Multi SVM output is a more compelling conclusion than all of the others.

**Fig. 6:**
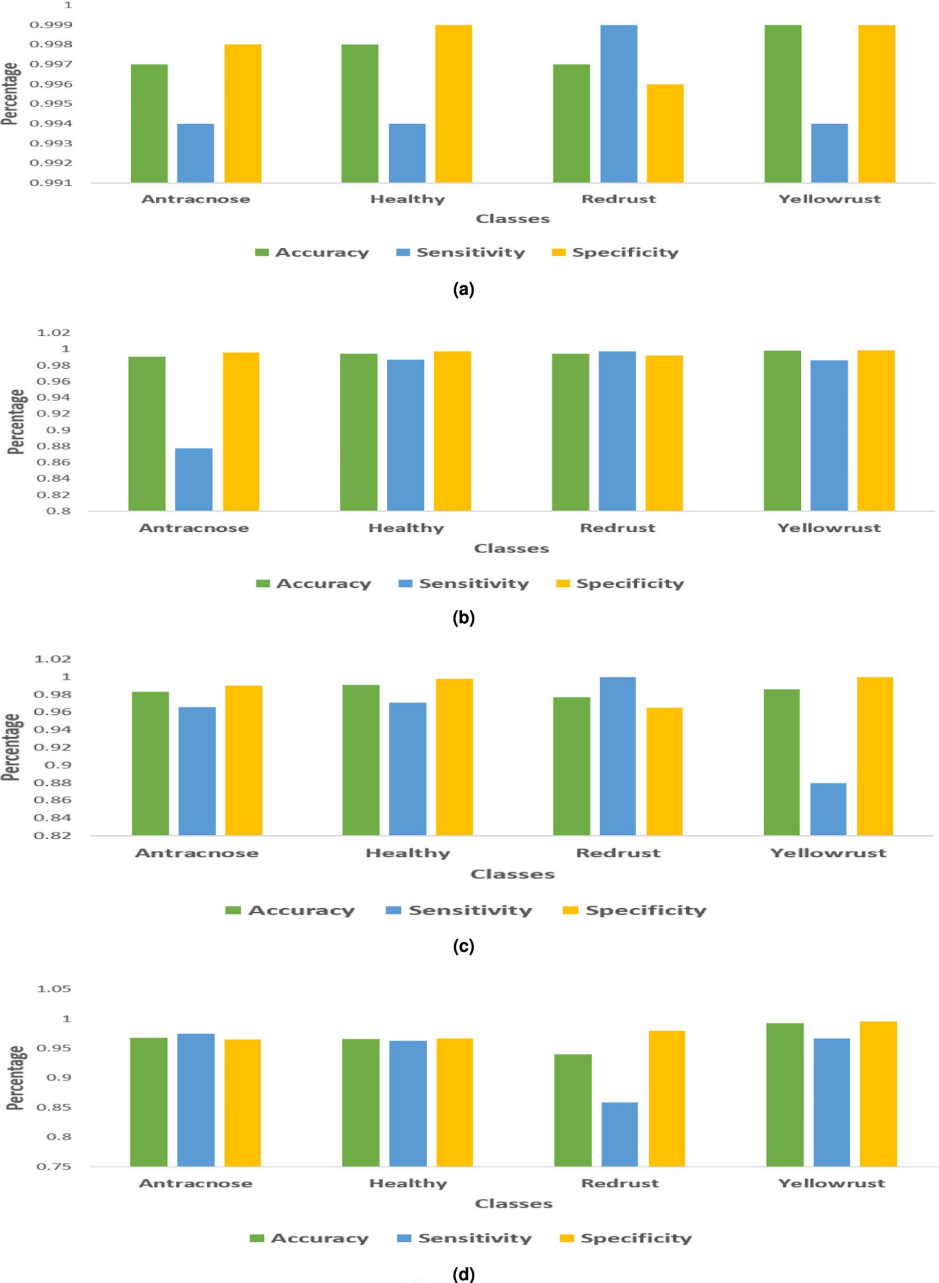
Performance metrics of SVM kernels with respect to GLCM, LPB features: (**a**) Linear, (**b**) RBF, (**c**) Quadratic (**d**) Cubic.

The result of the multiclass SVM classifier is shown in Fig. 6. The analysis of classification accuracy makes use of classifiers like SVM with four distinct kernel functions and multiclass SVM. The degree to which the calculated results closely resemble the right values is called accuracy. A comparative study employing an SVM-based approach was the most successful way to achieve consistent accuracy across fault categories in our challenge.

**Table 2:** Two-category categorization evaluation of performance.

Categorized in	Linear	RBF	Quadratic	Cubic
Healthy(%)	99.0	98.3	97.4	96.7
Diseased (%)	98.2	98.1	97.2	96.1

**Table 3:** SVM Classifier Performance: Two-Category Grading.

Categorized in	Healthy	Diseased
Healthy	9711	1413
Diseased	289	8587
Total	10000	10000
Accuracy (%)	99.0	98.2

## Metrics for evaluating classifier model performance

A contingency table, also known as a confusion matrix, provides a comprehensive view of classification results by tabulating the actual and predicted categories, displaying the specific results for each category^40^. By computing the confusion matrix^41^, a more nuanced understanding of the classification model’s strengths and weaknesses can be obtained. Various metrics are used to evaluate the efficacy of a methodology. Five conventional machine-learning classifiers and the proposed approach are compared using the following metrics: accuracy, precision, recall, and F1 score.

### Accuracy

It represents the percentage of samples that were correctly categorized across the entire dataset. It is calculated using the following formula:

$$\begin{aligned} Accuracy = \dfrac{\mathscr{T}\mathscr{N} + \mathscr{T}\mathscr{P}}{\mathscr{T}\mathscr{N} + \mathscr{T}\mathscr{P} + \mathscr{F}\mathscr{P} + \mathscr{F}\mathscr{N}}*100\% \end{aligned}$$

In terms of mathematics, accuracy looks like this:

$$\begin{aligned} Accuracy = \dfrac{Number~ of ~Correct~ Predictions}{Total~ Number~ of ~Predictions}\times 100 \end{aligned}$$

where,

- Number of Correct Predictions: The number of cases in which the model predicted the class label accurately.
- Total Number of Predictions: The overall quantity of cases that the model assessed.

### Sensitivity

In binary classification, sensitivity, also known as recall or the true positive rate, reflects the proportion of actual positive cases that the model correctly identifies. It is defined as the ratio of true positive detection to the total number of actual positive samples.

Sensitivity has the following mathematical definition:

$$\begin{aligned} Sensitivity = \dfrac{\mathscr{T}\mathscr{P}}{\mathscr{T}\mathscr{P} + \mathscr{F}\mathscr{N}}*100\% \end{aligned}$$

### Specificity

Sensitivity concentrates on positive cases, whereas specificity assesses how well a model identifies negative instances. The definition of specificity is:

$$\begin{aligned} Specificity = \dfrac{\mathscr{T}\mathscr{N}}{\mathscr{T}\mathscr{N} + \mathscr{F}\mathscr{P}}*100\% \end{aligned}$$

- Where $\mathscr{T}\mathscr{P}$ stands for True positive: If a common spot test yields a positive result, the plant is deemed to be infected. It’s called a true positive.
- A typical spot test result that is negative indicates that the crops are not infected with the crop disease, which is known as a true negative^42^.
- A false positive in a crop’s common spot test occurs when the test wrongly indicates the presence of the disease in crops that are actually disease-free. Conversely, a false negative occurs when the test fails to detect the presence of the disease in crops that are actually infected with common spot.

Positive Predictive Value ($\mathscr {P}\mathscr {P}\mathscr {V}$), also known as precision, calculates the proportion of true positive results among all positive predictions made by a diagnostic test. It measures the accuracy of the test in identifying true positives, with a higher PPV indicating better performance in correctly detecting positive instances.

$$\begin{aligned} \mathscr {P}\mathscr {P}\mathscr {V}=\dfrac{\mathscr{T}\mathscr{P}}{\mathscr{T}\mathscr{P}+\mathscr{F}\mathscr{P}}*100\% \end{aligned}$$

Negative Predictive Value ($\mathscr {N}\mathscr {P}\mathscr {V}$) measures the fraction of true negatives among all the instances that were predicted as negative. It indicates how effectively a test identifies true negatives, reflecting the accuracy of negative results.

$$\begin{aligned} NPV=\dfrac{\mathscr{T}\mathscr{N}}{\mathscr{T}\mathscr{N}+\mathscr{F}\mathscr{N}}*100\% \end{aligned}$$

The F-score, used to evaluate model performance, is the harmonic mean of precision and recall, adjusted by their relative importance.

$$\begin{aligned} F-Score=2* \dfrac{Precision*Sensitivity}{Precision+Sensitivity}*100\% \end{aligned}$$

**Table 4:** Performance metrics for classifiers across four stages, based on statistical features.

Kernel	Stage	TN	TP	FN	FP	Accuracy	Sensitivity	Specificity
Linear	Antracnose	6523	2561	15	10	0.997	0.994	0.998
	Healthy	6630	2461	13	5	0.998	0.994	0.999
	Red rust	6033	3051	3	21	0.997	0.999	0.996
	Yellow rust	8103	999	6	1	0.999	0.994	0.999
RBF	Antracnose	6509	254	35	24	0.991	0.878	0.996
	Healthy	6617	2444	30	18	0.994	0.997	0.987
	Red rust	6011	3046	8	44	0.994	0.997	0.992
	Yellow rust	8103	991	14	1	0.998	0.986	0.999
Quadratic	Antracnose	6470	2489	87	63	0.983	0.966	0.990
	Healthy	6628	2404	70	7	0.991	0.971	0.998
	Red rust	5848	3054	0	207	0.977	1	0.965
	Yellow rust	8104	885	120	0	0.986	0.880	1
Cubic	Antracnose	6309	2512	64	224	0.968	0.975	0.965
	Healthy	6388	2384	90	217	0.966	0.963	0.967
	Red rust	5939	2625	429	116	0.940	0.859	0.980
	Yellow rust	8075	972	33	29	0.993	0.967	0.996

## Result and discussion

In MATLAB 2023B, the suggested method for categorizing leaf diseases is implemented using the multi SVM setups described below. In the SVM classifier, the kernel function is set up with a box constraint value of 3 and a kernel scale of 4. The Bayesian optimization approach utilizes the expected improvement per second plus as its acquisition function. A total of 30 iterations are conducted across all kernels. The training duration for the four kernels (linear, RBF, quadratic, and cubic) are 143.15 seconds, 160.86 seconds, 83.12 seconds, and 92.77 seconds, respectively.

Although plant disease identification has seen remarkable outcomes using deep learning models such as Convolutional Neural Networks (CNNs) and Vision Transformers (ViTs), their effectiveness usually depends on having access to large-scale datasets to avoid overfitting. On a particular wheat rust dataset, for example^43^, used a hybrid CNN-ViT architecture and obtained an accuracy of 98.3%. Comparatively speaking, our composite dataset consists of 9,111 images from various crop types; it is small in terms of size but diverse in substance. CNN-based methods are also frequently computationally demanding, which may limit their applicability for implementation in actual agricultural environments with limited hardware capabilities. In contrast, the SVM-based method presented in this study delivers both computational efficiency and model transparency. It achieves 99.0% accuracy using streamlined preprocessing and handcrafted feature extraction. This work acts as a baseline to direct and assess further deep learning advancements on this dataset.

The results in Table 5 are reported as mean ± standard deviation across the five cross-validation folds. This approach demonstrates that the high accuracy values were consistent across different data partitions. For example, the linear kernel achieved 99.0% ± 0.3 accuracy, while the RBF kernel achieved 98.6% ± 0.4, showing only minor variation between folds. These low variances confirm the robustness and reproducibility of the proposed pipeline.

**Table 5:** Performance metrics (mean± standard deviation) of kernel classifiers across 5-fold cross-validation.

Performance metrics	Linear	RBF	Quadratic	Cubic
Accuracy (%)	99.0± 0.3	98.6±0.4	97.0±0.6	93.2± 0.8
Sensitivity (%)	0.995±0.2	0.962± 0.5	0.954± 0.7	0.941± 0.9
Specificity (%)	0.998±0.1	0.996±0.3	0.988± 0.5	0.977±0.7
PPV (%)	0.996±0.2	0.972±0.4	0.977±0.6	0.941± 0.8
NPV(%)	0.998±0.1	0.996± 0.3	0.990±0.5	0.968±0.7
F-score (%)	1.492±0.2	0.966±0.4	0.963±0.6	0.939±0.8

### Evaluation of multi (Shape and texture (GLGCM, LBP)) feature classification performance

**Fig. 7:**
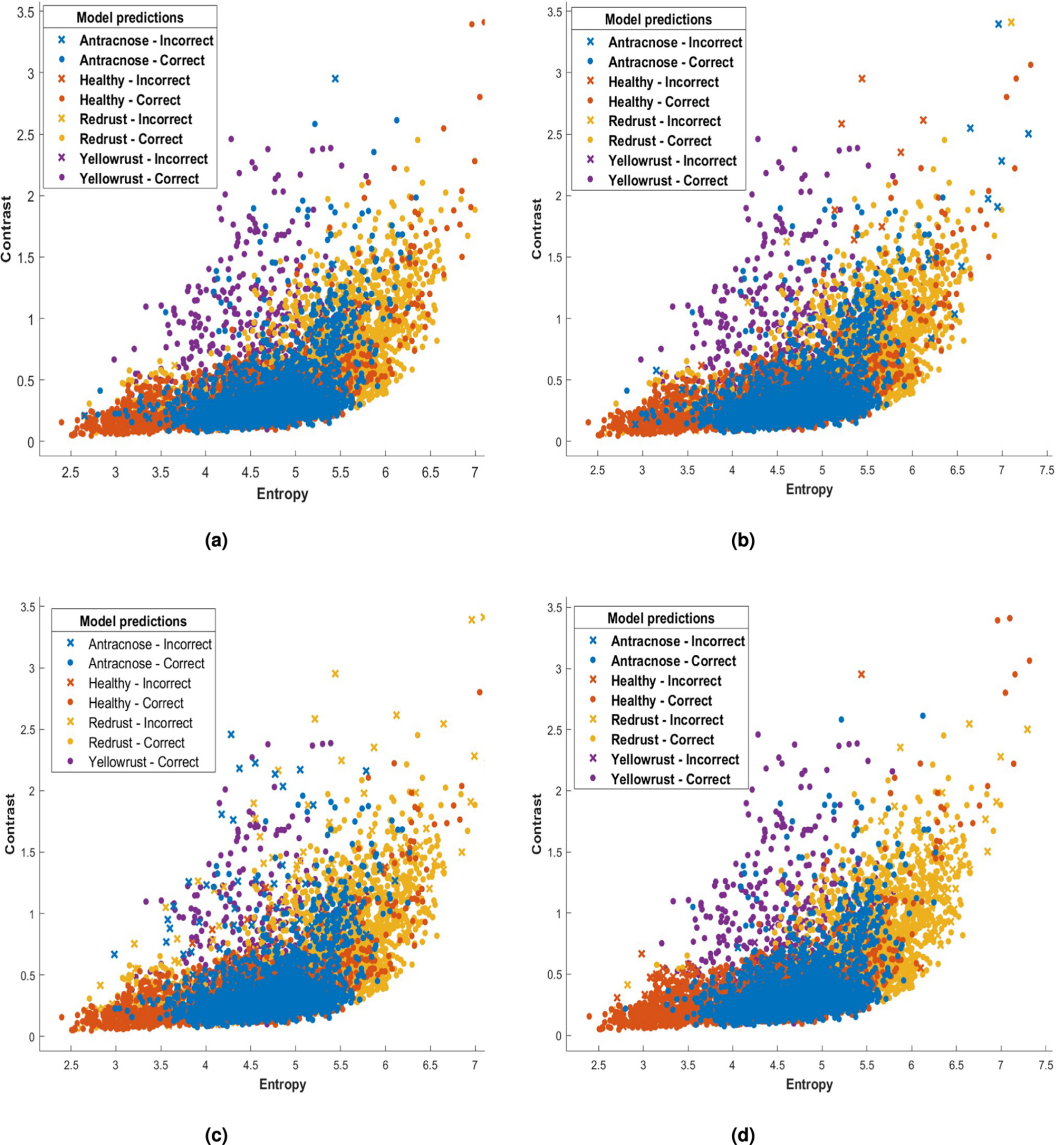
Scatter plot of SVM classifier with kernels (**a**) Linear (**b**) RBF (**c**) Quadratic (**d**) Cubic.

A scatter plot showing each classifier’s stage classifications based on contrast and entropy measurements is shown in Fig. 7. The plot highlights significant variability in the data throughout all stages of plant disease detection. The dataset features four categories: healthy, anthracnose, red rust, and yellow rust. In the scatter plot, the predicted class is labeled as PPV and FDR, where PPV represents positive predictive value and FDR refers to false discovery rate.

The disparities in classifier performance underscore the importance of selecting an appropriate model that is customized to the distinct characteristics of the dataset. Classification accuracy may be increased by modifying hyperparameters and researching cutting-edge tactics like ensemble approaches. Furthermore, the classifiers’ resistance to misclassifications may be improved by adding more features or using data enrichment techniques.

**Fig. 8:**
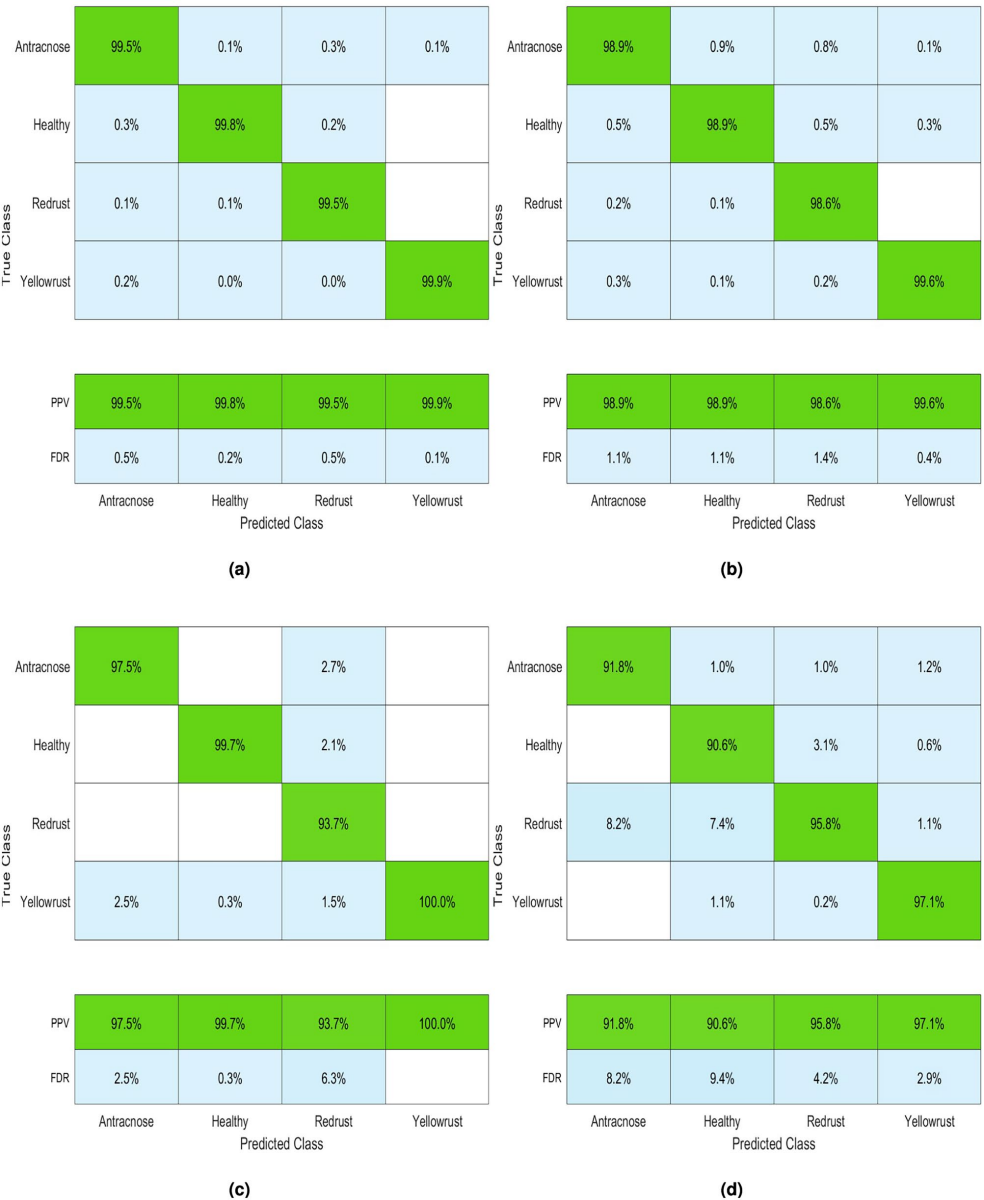
Confusion matrix of SVM classifier with kernel (**a**) Linear (**b**) RBF (**c**) Quadratic (**d**) Cubic.

Table 4 presents the classification accuracy, sensitivity, and specificity for each stage of the subject, assessed using four different classifiers. The linear kernel achieved the highest performance, correctly predicting yellow rust in nearly all cases with only 0.1% misclassification. Anthracnose and red rust showed minor errors, but overall accuracy remained 99%. The RBF kernel performed slightly lower (98.6%), while the quadratic kernel identified yellow rust well but struggled with other classes (97.0%). The cubic kernel showed the weakest performance, with frequent misclassifications and 93.2% accuracy (Table 5). Additionally, Fig. 8 depicts the overall performance of the classifiers for each kernel using a confusion matrix. This figure compares the predicted outcomes with the actual results.

**Fig. 9:**
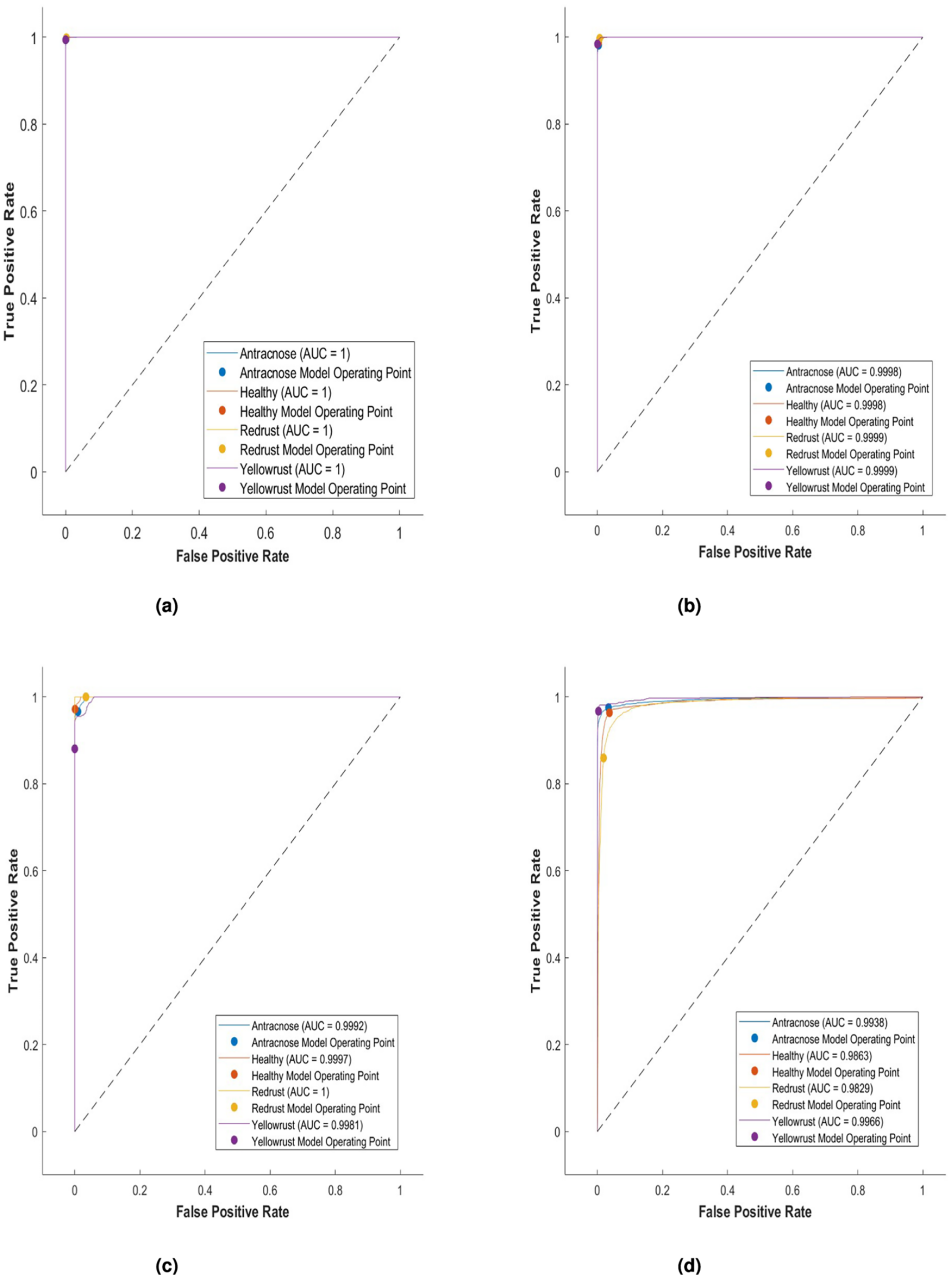
ROC of SVM with kernel (**a**) Linear (**b**) RBF (**c**) Quadratic (**d**) Cubic.

The Receiver Operating Characteristic (ROC) curve illustrates the trade-off between sensitivity (true positive rate) and specificity (1–false positive rate) across various thresholds. The Area Under the Curve (AUC) reflects the overall separability of classes, where a value of 1.0 indicates perfect classification and 0.5 indicates random guessing. In this study, Fig. 9 presents the ROC–AUC values for the four kernels, demonstrating that the classifiers effectively distinguish among the different disease classes using multiple features.

**Table 6:** Comparison Table.

Reference	Classifier	Features	Accuracy
^8^	ANN, SVM, KNN, NB, and CNN	shape	91.5% & 90%
^10^	ANN, KNN, RF, and SVM	GLCM	94%
^11^	KNN and Max Voting	Temperature data	91%
^13^	DAE, DNN, and ANN	Deep-CNN	91%
^14^	CNNC and IKNN	Gradient and extended gradient features	98.07%, 96.60%
^15^	Moth Optimization based Deep Neural Network (MO-DNN)	MSO	0.973%
^2^	SVM, CNN, and CNN&ViT	CNN and CNN&ViT automated, SVM handcrafted or pre-trained	91.7%,95.97% & 98.0%
^19^	k-NN, SVM, ANN, and RF	Color (Lab* components) and texture (LBP)	Disease type 97.4%, Degree of infection 91.0%
Proposed model	SVM-Linear	GLCM, LBP, Shape	99.0%

In conclusion, this study is innovative in the way it systematically combines and evaluates the pipeline’s separate components, which include bilateral filtering, Y*Cb*Cr + GraphCut segmentation, GLCM, LBP, and multiclass SVM. These components are not new in and of themselves. Using a carefully selected, multi-crop dataset, this work benchmarks the performance of various SVM kernels, offering a cohesive and organized method. A solid basis for upcoming comparisons with deep learning models is established by the use of stratified 5-fold cross-validation and a variety of assessment criteria, which guarantee reproducibility.

## Limitation of the study

The suggested approach performed well on the curated multi-crop dataset; however, there are a few things to keep in mind. First, rather than being taken in actual agricultural fields, the images were taken from publicly accessible repositories. Consequently, elements like erratic lighting, occlusions, and intricate field backdrops were not adequately depicted. Second, the model’s versatility may be limited when applied to extremely different or unknown datasets because it mostly relies on manually created texture features (GLCM and LBP). Finally, while four different SVM kernels were systematically compared, the framework’s scalability to very large datasets and its suitability for real-time deployment remain to be validated. Future work will address these issues by incorporating field-collected images, combining handcrafted and deep learning–based features, and testing the model under real-world conditions.

## Conclusion and future research work

A machine learning-based system for crop leaf disease identification was presented in this paper, with a focus on four classes: anthracnose, yellow rust, red rust, and healthy. GraphCut segmentation was used to isolate sick regions, bilateral filtering was used to reduce noise, and hybrid texture-based feature extraction using GLCM and LBP was used to analyze a curated and enhanced dataset of 9,111 multi-crop images. Using a multiclass SVM and four kernel functions (linear, RBF, quadratic, and cubic), classification was carried out.

The experimental results demonstrated that the linear SVM kernel achieved the best performance, with 99.0% accuracy, 98.6% precision, 98.7% recall, and a 98.6% F1-score, outperforming previously reported SVM-based approaches. These results demonstrate that well-crafted lightweight models can achieve high accuracy and robustness comparable to deep learning techniques while requiring fewer computational resources, confirming the significance of integrating preprocessing, segmentation, and kernel evaluation within a single pipeline. The comparative analysis with existing approaches is summarized in Table 6, further highlighting the strength of our framework. This has real-world implications for creating accessible, affordable, and scalable plant disease detection technologies that promote food security and early intervention.

Nonetheless, certain limitations remain. The studies were all carried out on publicly accessible datasets under controlled conditions, therefore issues like sensor noise, complicated field backgrounds, and fluctuating illumination were not entirely resolved.

The approach will be validated using in-field datasets in future studies, its application to other crop-disease combinations will be expanded, and integration with drone-based aerial photography will be investigated. Additionally, evaluating the trade-offs between more resource-intensive deep learning systems and lightweight SVM-based pipelines will be made easier by benchmarking against cutting-edge deep learning models (such as ResNet, MobileNet, and Vision Transformers). Longer term, integrating these frameworks into real-time decision-support systems can give farmers practical advice on how to lower production losses and advance sustainable farming methods.

## Data Availability

The datasets used and/or analysed during the current study available from the corresponding author on reasonable request.

## References

[1] Khan, I., Sohail, S. S., Madsen, D. Ø. & Khare, B. K. Deep transfer learning for fine-grained maize leaf disease classification. *J. Agric. Food Res.* **16**, (2024).

[2] Akinosun, T. & Nibouche, O. Ai-based crop disease detection: Evaluation of wheat rust disease detection and classification using deep learning and machine learning approaches. In *Proceedings of the 2023 31st Irish Conference on Artificial Intelligence and Cognitive Science (AICS)*, 1–4 (IEEE, 2023).

[3] Ahmed, I. & Yadav, P. K. Plant disease detection using machine learning approaches. *Expert. Syst.* **40**, (2023).

[4] Kumar, M., Kumar, A. & Palaparthy, V. S. Soil sensors-based prediction system for plant diseases using exploratory data analysis and machine learning. *IEEE Sensors J.* **21**, 17455–17468 (2020).

[5] Neelakantan, P. Analyzing the best machine learning algorithm for plant disease classification. *Mater. Today: Proc.* (2021).

[6] Hou, C. et al. Recognition of early blight and late blight diseases on potato leaves based on graph cut segmentation. *J. Agric. Food Res.* **5**, (2021).

[7] Pantazi, X. E., Moshou, D. & Tamouridou, A. A. Automated leaf disease detection in different crop species through image features analysis and one class classifiers. *Comput. Electron. Agric.* **156**, 96–104 (2019).

[8] Kasinathan, T., Singaraju, D. & Uyyala, S. R. Insect classification and detection in field crops using modern machine learning techniques. *Inf. Process. Agric.* **8**, 446–457 (2021).

[9] Pallathadka, H. et al. Application of machine learning techniques in rice leaf disease detection. *Mater. Today: Proc.* **51**, 2277–2280 (2022).

[10] Nyasulu, C. et al. A comparative study of machine learning-based classification of tomato fungal diseases: Application of glcm texture features. *Heliyon* **9** (2023). 10.1016/j.heliyon.2023.e21697 PMC10656238 38027996

[11] Nalini, T. & Rama, A. Impact of temperature condition in crop disease analyzing using machine learning algorithm. *Meas. Sensors* **24**, 100408 (2022).

[12] Ahmed, I. & Yadav, P. K. A systematic analysis of machine learning and deep learning based approaches for identifying and diagnosing plant diseases. *Sustain. Oper. Comput.* **4**, 96–104 (2023).

[13] Sobiyaa, P., Jayareka, K. S., Maheshkumar, K., Naveena, S. & Rao, K. S. Paddy disease classification using machine learning technique. *Mater. Today: Proc.* **64**, 883–887 (2022).

[14] Shantkumari, M. & Uma, S. V. Machine learning techniques implementation for detection of grape leaf disease. *Multimed. Tools Appl.* **82**, 30709–30731 (2023).

[15] Suresh & Seetharaman, K. Real-time automatic detection and classification of groundnut leaf disease using hybrid machine learning techniques. *Multimed. Tools Appl.* **82**, 1935–1963 (2023).

[16] Upadhyay, N. & Bhargava, S. Artificial intelligence in agriculture: applications, approaches, and adversities across pre-harvesting, harvesting, and post-harvesting phases. *Environ. Monit. Assess.* **196**, 12790 (2025).

[17] Upadhyay, N. & Gupta, A. Seglearner: A segmentation based approach for predicting disease severity in infected leaves. *Discov. Artif. Intell.* **5**, 264 (2025).

[18] Kiran, A., Naik, S. L., Raj, M. S. & Palvadi, S. K. Plant disease detection using image processing with machine learning. In *2023 4th International Conference on Electronics and Sustainable Communication Systems (ICESC)*, 1590–1595 (IEEE, 2023).

[19] Hou, C. et al. Recognition of early blight and late blight diseases on potato leaves based on graph cut segmentation. *J. Agric. Food Res.* **5**, (2021).

[20] Kaagle. Crop pest and disease detection. https://www.kaggle.com/datasets/nirmalsankalana/crop-pest-and-disease-detection (n.d).

[21] Kaagle. 20k multi-class crop disease images. https://www.kaggle.com/datasets/jawadali1045/20k-multi-class-crop-disease-images (n.d).

[22] Tomasi, C. & Manduchi, R. Bilateral filtering for gray and color images. *Proc. Sixth Int. Conf. on Comput. Vis.* **1998**, 839–846 (1998).

[23] Ashwinkumar, S., Rajagopal, S., Manimaran, V. & Jegajothi, B. Automated plant leaf disease detection and classification using optimal mobilenet based convolutional neural networks. *Mater. Today: Proc.* **51**, 480–487 (2022).

[24] Entuni, C. J., Zulcaffle, T. M. A., Kipli, K. & Kurugollu, F. Severity estimation of plant leaf diseases using segmentation method. *Appl. Sci. Eng. Prog.* **14**, 108–119 (2021).

[25] Boykov, Y. & Jolly, M.-P. Interactive graph cuts for optimal boundary & region segmentation of objects in n-d images. *Proc. IEEE Int. Conf. on Comput. Vis.* **1**, 105–112 (2001).

[26] Vicente, S., Kolmogorov, V. & Rother, C. Graph cut based image segmentation with connectivity priors. In *2008 IEEE Conference on Computer Vision and Pattern Recognition*, 1–8 (IEEE, 2008).

[27] Rapaka, A. An intelligent convolution based graph cut segmentation for potato leaf disease severity prediction. *Multimed. Tools Appl.* **83**, 32765–32787 (2024).

[28] Haralick, R. M., Shanmugam, K. & Dinstein, I. Textural features for image classification. *IEEE Transactions on Syst. Man, Cybern.* **SMC-3**, 610–621 (1973).

[29] Ojala, T., Pietikäinen, M. & Harwood, D. A comparative study of texture measures with classification based on feature distributions. *Pattern Recognit.* **29**, 51–59 (1996).

[30] Gayathri Devi, T. & Neelamegam, P. J. C. C. Image processing based rice plant leaves diseases in thanjavur, tamilnadu. *Clust. Comput.* **22**, 13415–13428 (2019).

[31] Khan, R., Ud Din, N., Zaman, A. & Huang, B. Automated tomato leaf disease detection using image processing: An svm-based approach with glcm and sift features. *J. Eng.* **2024**, 9918296 (2024).

[32] Lladó, X., Oliver, A., Freixenet, J., Martí, R. & Martí, J. A textural approach for mass false positive reduction in mammography. *Comput. Med. Imaging Graph.* **33**, 415–422 (2009). 19406614 10.1016/j.compmedimag.2009.03.007

[33] Arifin, M., Yuniarti, A. & Suciati, N. Cucumber disease image classification with a model combining lbp and vgg-16 features. *Int. J. Robotics & Control. Syst* **4** (2024).

[34] Elkurdi, A., Soufian, M. & Nefti-Meziani, S. Gait speeds classifications by supervised modulation based machine-learning using kinect camera. *Med. Res. Innov.* **2** (2018).

[35] Moon, T. K. *Error correction coding: mathematical methods and algorithms* (John Wiley & Sons, 2020).

[36] Picard, D. Multiple locally linear kernel machines. arXiv preprint arXiv:2401.09629 (2024).

[37] Anisa, Y., Erika, W. & Azmi, F. Enhancing student performance prediction using a combined svm-radial basis function approach. *Int. J. Innov. Res. Comput. Sci. & Technol.* **12**, 1–5 (2024).

[38] Ratha, A. K., Behera, S. K., Devi, A. G., Barpanda, N. K. & Sethy, P. K. Optimizing precision agriculture: Bayesian-enhanced papaya (carica papaya l.) fruit disease classification via cubic svm and resnet-101 deep features. *J. Intell. & Fuzzy Syst.* 1–17. Preprint.

[39] Lin, F., Fang, S. C., Fang, X., Gao, Z. & Luo, J. A distributionally robust chance-constrained kernel-free quadratic surface support vector machine. *Eur. J. Oper. Res.* **316**, 46–60 (2024).

[40] Karthik, C. & Ulaganathan, N. Application for plant’s leaf disease detection using deep learning techniques. *Int. Res. J. Eng. Technol. (IRJET)* **7**, 1–7 (2020).

[41] Agarwal, M., Singh, A., Arjaria, S., Sinha, A. & Gupta, S. Toled: Tomato leaf disease detection using convolution neural network. *Procedia Comput. Sci.* **167**, 293–301 (2020).

[42] Argüeso, D. et al. Few-shot learning approach for plant disease classification using images taken in the field. *Comput. Electron. Agric.* **175**, (2020).

[43] Akinosun, T. & Nibouche, O. Ai-based crop disease detection: Evaluation of wheat rust disease detection and classification using deep learning and machine learning approaches. In *2023 31st Irish Conference on Artificial Intelligence and Cognitive Science (AICS)*, 1–4 (IEEE, 2023).

